# Incidence and Determinants of Tuberculosis among Adults Initiating Antiretroviral Therapy – Mozambique, 2004–2008

**DOI:** 10.1371/journal.pone.0054665

**Published:** 2013-01-22

**Authors:** Andrew F. Auld, Francisco Mbofana, Ray W. Shiraishi, Charity Alfredo, Mauro Sanchez, Tedd V. Ellerbrock, Lisa J. Nelson

**Affiliations:** 1 Division of Global HIV/AIDS (DGHA), Center for Global Health (CGH), Centers for Disease Control and Prevention, Atlanta, Georgia, United States of America; 2 Insituto Nacional de Saúde, Ministry of Health, Maputo, Mozambique; 3 Division of Global HIV/AIDS (DGHA), Center for Global Health (CGH), Centers for Disease Control and Prevention, Maputo, Mozambique; 4 Department of Public Health, School of Health Sciences, University of Brasilia, Brasilia, Brazil; National Institute of Allergy and Infectious Diseases, United States of America

## Abstract

**Background:**

In Mozambique, tuberculosis (TB) is thought to be the most common cause of death among antiretroviral therapy (ART) enrollees. Monitoring proportions of enrollees screened for TB, and incidence and determinants of TB during ART can help clinicians and program managers identify program improvement opportunities.

**Methodology/Principal Findings:**

We conducted a retrospective cohort study among a nationally representative sample of the 79,500 adults (>14 years old) initiating ART during 2004–2007 to estimate clinician compliance with TB screening guidelines, factors associated with active TB at ART initiation, and incidence and predictors of documented TB during ART follow-up. Of 94 sites enrolling >50 adults on ART, 30 were selected using probability-proportional-to-size sampling; 2,596 medical records at these sites were randomly selected for abstraction and analysis. At ART initiation, median age of patients was 34, 62% were female, median baseline CD4^+^ T-cell count was 153/µL, and 11% were taking TB treatment. Proportions of records with TB screening documentation before ART initiation improved from 31% to 66% during 2004–2007 (p<0.001). TB screening compliance varied widely by ART clinic [n = 30, 2%–98% (p<0.001)] and supporting non-Governmental Organization (NGO) [n = 7, 27%–83% (p<0.001)]. Receiving TB treatment at ART enrollment was associated with male sex (p<0.001), weight <45 kg (p<0.001) and CD4<50/µL (p = 0.001). Isoniazid preventive therapy (IPT) was prescribed to <1% of ART enrollees not taking TB treatment. TB incidence during ART was 2.32 cases per 100 person-years. Factors associated with TB incidence included adherence to ART <95% (AHR 2.06; 95% CI, 1.32–3.21).

**Conclusion:**

Variations in TB screening by clinic and NGO may reflect differing investments in TB screening activities. Future scale-up should target under-performing clinics. Scale-up of TB screening at ART initiation, IPT, and ART adherence interventions could significantly reduce incident TB during ART.

## Introduction

In Mozambique, rising adult HIV prevalence from about 2% in 1990 to 11.5% in 2009 [1] has fueled the country’s tuberculosis (TB) epidemic. During 1990–2010, TB case notification rates increased from 401/100,000 population to 544/100,000 population (a 36% increase), driven mainly by increases in TB-HIV co-infection rates, from 51/100,000 population to 330/100,000 population (a 647% increase) [2]. With increases in TB-HIV incidence, and high mortality among HIV-infected patients with undiagnosed TB or diagnosed TB but no access to antiretroviral therapy (ART), TB is thought to account for nearly a quarter of Mozambique’s national HIV/AIDS mortality [3].

Although ART reduces TB acquisition risk among HIV-infected persons by about 67% [4], high TB incidence is commonly observed immediately after ART start [5]. Even after years of therapy, TB incidence can be twice as high as that in the general population [6]. Understanding expected TB morbidity during ART is important for TB-HIV program managers for planning purposes and for clinicians involved in patient management [7].

Autopsy studies suggest that undiagnosed TB is a common cause of mortality among HIV-infected patients [8]–[11]. Therefore, monitoring the proportions of patients correctly screened for TB prior to ART start is important for TB-HIV program monitoring. Similarly, since late 2006, the Mozambican Ministry of Health (MOH) began recommending isoniazid preventive therapy (IPT) for HIV-infected persons when active TB has been excluded, and monitoring IPT uptake is an important TB-HIV program monitoring activity.

Investigating risk factors associated with prevalent TB (active TB at ART enrollment) and incident TB during ART follow-up is important for clinicians to identify patients at risk, and can help program managers identify program improvement opportunities.

Therefore, we conducted a retrospective cohort study among a nationally representative sample of the 79,500 adult ART patients who initiated therapy during 2004–2007 in Mozambique to describe TB screening practices, the prevalence of active TB at ART enrollment, IPT uptake among patients not taking TB treatment at ART start, TB incidence during ART follow-up, and factors associated with prevalent and incident TB.

## Methods

### Ethics Approval

This study was approved by the Institutional Review Board (IRB) of the United States Centers for Disease Control and Prevention (CDC) and the Mozambican MOH Ethics Review Committee (*Ministerio da Saude Comite Nacional de Bioetica para a Saude*). Both review boards approved the consent procedures. Patient informed consent was not required as only routine, anonymous, operational monitoring data were collected and analyzed.

### Eligibility for ART

During 2004–2007, patients diagnosed with World Health Organization (WHO) stage IV HIV disease, stage III disease with CD4^+^ T-cell (CD4) counts <350/µL, or stage I or II disease with CD4 counts <200/µL, were eligible for ART [12]. First-line ART regimens included two nucleoside reverse-transcriptase inhibitors (NRTI) and a non-nucleoside reverse transcriptase inhibitor (NNRTI). Second-line regimens contained a protease inhibitor and two new NRTIs.

### TB Screening and Diagnosis

Mozambique guidelines recommend that HIV-infected adults be screened for TB at all HIV clinic visits, including the ART enrolment visit. However, during 2004–2006, national ART guidelines did not specify recommended TB screening questions to identify TB suspects. In 2007, a new TB screening instrument was introduced which screened for: (1) cough for >3 weeks, (2) any cough with blood, (3) night sweats >3 weeks, (4) fever >3 weeks, (5) weight loss >3 weeks, and (6) any TB contacts [13]. From 2007 onwards, if one or more of these six TB symptoms were positive, the patient was considered a TB suspect. For all TB suspects, regardless of how they were identified during 2004–2008, two on-the-spot sputum samples were sent to the laboratory for Ziehl-Neelson sputum microscopy. For smear-negative TB suspects, chest X-ray was recommended. TB culture, available at two central Maputo laboratories [2], was seldom requested by clinicians due to sample transport barriers, and long wait times for results. If extra-pulmonary TB was suspected, lymph node aspiration, lumbar puncture, and abdominal ultrasound were additional diagnostic tests available at larger facilities. Usually TB treatment was managed at TB clinics, separate from ART clinics.

### Patient Monitoring

Mozambique guidelines recommend adults, who are newly initiating ART, be seen frequently initially (at 2 weeks, monthly for 3 months, and quarterly thereafter) until stable on therapy and then at least every 6 months, for weight measurements, clinical staging, TB screening, hemoglobin measurements, and CD4 count testing. At each visit, standard Ministry of Health (MOH) recommended medical records are completed. During 2004–2008, patients collected medications monthly from clinic pharmacies.

### Study Design and Population

This was a secondary analysis of a retrospective cohort study designed to assess incidence and predictors of attrition [death, loss to follow-up (LTFU), or stopping ART], among a nationally representative sample of adult ART patients (>15 at ART start), who initiated ART during 2004–2007 in Mozambique [14]. This analysis investigates factors associated with active TB at ART initiation (prevalent TB) and incident TB during ART follow-up, among those not taking TB treatment at ART initiation.

### Treatment Outcome Measures

Any patient taking TB treatment (for pulmonary or extra-pulmonary TB) at ART initiation was considered to have prevalent TB at ART start. The first occurrence of TB treatment (for pulmonary or extra-pulmonary TB) among adults, not on TB treatment at ART initiation, was considered incident TB.

### Exposure Variables

Variables routinely collected on the MOH-recommended ART records, including age at ART start, sex, previous TB diagnoses, weight, WHO stage, CD4 count, hemoglobin, and co-trimoxazole prescription, were assessed as possible risk factors for prevalent and incident TB. Laboratory machines and methods for measuring CD4 counts and hemoglobin in Mozambique’s ART program, have been previously described [14]. Similar to other cohort studies in resource-constrained settings [15], adherence to ART was estimated by measuring timeliness of patient visits to scheduled medicine pick-up appointments at clinic-based pharmacies during months 0–6 of ART [16]–[18].

### Sample Size

To answer the original primary study question concerning attrition with the desired precision, a sample size of >1,200 patient records was needed to achieve a 95% confidence interval (CI) of +3.0% or smaller around a 25% [19] 6-month attrition estimate, assuming a design effect of 1.5. A target sample size of 2,600 medical records was chosen to meet the needs of a concurrent cost-effectiveness study.

### Sampling

MOH-reported data from December 31, 2006, were used to define the clinic sample frame. By December 2006, 43,295 adults had initiated ART at 152 clinics. Clinics with <50 adults enrollees by December 31, 2006, were excluded from the sample frame to improve study feasibility, resulting in 58 clinics, with 1,061 adult ART enrollees, being excluded. Of the 94 clinics enrolling >50 adults on ART, 12 with >1,000 enrollees were selected with certainty, while 18 with 50–1,000 enrollees were selected using probability-proportional-to-size sampling; we aimed to randomly sample 2,600 medical records of adults who initiated ART during 2004–2007 at these 30 sites. To meet the needs of a concurrent cost-effectiveness study, 150 medical records were sampled at each of 11 sites involved in this concurrent study (1,650 records); at the remaining 19 sites, 50 medical records were randomly selected per site (950 records). At each of the selected 30 sites, the desired sample size of eligible medical records was randomly selected from paper or electronic ART patient registers. If the paper medical record of a selected patient was missing, the team searched in all likely locations at the clinic (e.g. pharmacy, consulting room, or social worker’s office). If not found, a replacement medical record was randomly selected. Data from selected records were abstracted in November 2008.

### Analytic Methods

Data were analyzed using SAS 9.2 (SAS Institute Inc., Cary, NC), and STATA 10 (StataCorp, 2009, Stata Statistical Software, Release 10, College Station, TX). Data were weighted and survey design controlled for, during analysis.

Multiple imputation with chained equations was used to impute missing baseline demographic and clinical data for covariates of interest [20]. The ice [21]–[23] procedure in Stata was used to create 20 imputed datasets. The imputation model included incident TB as the event indicator, all study variables, and the Nelson-Aalen estimate of cumulative hazard [24]. Missing data were assumed missing at random (MAR) and all patients had complete time-to-event data (i.e., all patients had an ART initiation date, outcome status by November 2008, and date of outcome).

Associations between baseline covariates and active TB at ART start (prevalent TB) were assessed using bivariate logistic regression, with random effects specified for each facility. The sensitivity of the quadrature approximation was checked for random effects logistic regression models. Estimates across imputed datasets were combined according to Rubin’s rules [20] using the mim procedure in Stata [25].

Because medical records did not indicate timing of TB treatment cessation for those patients taking TB treatment at ART enrollment, time-to-event analysis to assess incidence and determinants of TB during ART follow-up was restricted to those patients considered TB-free at ART enrollment. The event of interest was first incidence of pulmonary or extra-pulmonary TB. Patients not documented to have been diagnosed with TB during ART follow-up were censored at the most recent visit, date of death if death occurred, or date of transfer if transferred. Cox proportional hazards regression models were used to estimate adjusted hazard ratios and 95% confidence intervals (CI). The proportional hazards assumption was assessed using visual methods and the Grambsch and Therneau test [26]. Estimates were combined across the imputed datasets according to Rubin’s rules [20] using the mim procedure in Stata [25].

Kaplan-Meier curves were used to examine cumulative probability of remaining undiagnosed with TB over time stratified by baseline variables.

## Results

Data for 2,596 adult patients enrolled on ART during 2004–2007 were abstracted and analyzed. At ART initiation, median age was 34, 62% were female, and median CD4 count was 153/µL (IQR, 76–231/µL), being lower for males than females (139/µL vs. 159/µL, p<0.01). Prior completed TB treatment was documented for 20% of patients, while 11% of patients (95% CI, 9–13%), had active TB at ART start (prevalent TB) (table 1).

**Table 1:** Associations between Patient Characteristics and Prevalence of Active TB at ART Initiation.

	All Patients at Enrollment (n = 2,596)						Receiving TB Treatment at ART Enrolment(n = 271, imputed)		Not Receiving TB Treatment at ART Enrolment(n = 2,325, imputed)		P-value**
	Original*				Imputed						
	N	N	%/median	IQR/CI	%/median	IQR/CI	Median/%	IQR/CI	Median/%	IQR/CI	
**Age at enrolment**											
Median (IQR) year	2,596	2,596	**34**	(28–42)	**34**	(28–42)	**36**	(29–42)	**34**	(28–42)	0.513
**Sex**											
Female	1,576	2,596	**62%**	(59–65)	**62%**	(59–65)	**52%**	(48–56)	**63%**	(60–66)	**0.003**
Male	1,020	2,596	**38%**	(35–41)	**38%**	(35–41)	**48%**	(44–52)	**37%**	(34–40)	
**Marital Status**											
Civil union|married	1,152	2,363	**47%**	(44–51)	**47%**	(43–51)	**53%**	(47–59)	**53%**	(49–57)	0.226
Single|widowed	1,211	2,363	**53%**	(49–56)	**53%**	(49–57)	**47%**	(41–53)	**47%**	(43–51)	
Missing	233	2,596	**9%**								
**Employment**											
Employed	992	2,268	**46%**	(40–51)	**45%**	(40–51)	**47%**	(42–51)	**45%**	(39–51)	0.764
Student	107	2,268	**4%**	(3–5)	**4%**	(3–5)	**6%**	(3–8)	**4%**	(3–5)	
Unemployed	1,169	2,268	**50%**	(45–56)	**51%**	(45–56)	**47%**	(43–51)	**51%**	(45–57)	
Missing	328	2,596	**13%**								
**WHO Stage**											
Stage I/II	619	1,617	**37%**	(32–42)	**41%**	(35–47)	**6%**	(3–9)	**45%**	(39–52)	**<0.001**
Stage III	739	1,617	**47%**	(43–52)	**44%**	(38–49)	**61%**	(53–70)	**42%**	(36–47)	
Stage IV	259	1,617	**16%**	(13–18)	**15%**	(12–18)	**33%**	(24–42)	**13%**	(10–16)	
Missing	979	2,596	**38%**								
**Weight**											
>60 kg	470	2,061	**17%**	(14–20)	**18%**	(15–21)	**13%**	(9–17)	**27%**	(23–30)	**<0.001**
45–60 kg	1,224	2,061	**59%**	(56–61)	**57%**	(53–60)	**52%**	(45–59)	**57%**	(54–60)	
<45 kg	367	2,061	**24%**	(21–27)	**25%**	(22–29)	**34%**	(29–40)	**16%**	(13–19)	
Missing	535	2,596	**21%**								
**CD4 Count**											
≥200/µL	754	2,254	**34%**	(28–39)	**33%**	(28–38)	**20%**	(15–25)	**35%**	(29–41)	**0.001**
50 – <200/µL	1,144	2,254	**50%**	(46–54)	**50%**	(46–54)	**54%**	(49–59)	**50%**	(46–54)	
<50/µL	356	2,254	**16%**	(14–18)	**17%**	(14–19)	**26%**	(23–29)	**15%**	(13–17)	
Missing	342	2,596	**13%**								
**Hemoglobin**											
≥8 g/dL	1,664	1,899	**87%**	(85–88)	**86%**	(84–88)	**75%**	(69–81)	**87%**	(85–90)	**<0.001**
<8 g/dL	235	1,899	**13%**	(12–15)	**14%**	(12–16)	**25%**	(19–31)	**13%**	(10–15)	
Missing	697	2,596	**27%**								
**Co-trimoxazole**											
Prescribed CTX	821	2,596	**31%**	(26–36)	**31%**	(26–36)	**33%**	(26–40)	**31%**	(26–35)	0.819
Not prescribed CTX	1775	2,596	**69%**	(64–74)	**69%**	(64–74)	**67%**	(60–74)	**69%**	(65–74)	
**Adherence**											
≥95% adherent	1,263	1,860	**73%**	(67–79)	**72%**	(66–78)	**76%**	(69–84)	**72%**	(66–78)	0.958
<95% adherent	597	1,860	**27%**	(21–33)	**28%**	(22–34)	**24%**	(66–78)	**28%**	(22–34)	
Missing	736	2,596	**28%**								
**Site Size**											
>1,000	487	2,596	**23%**	(10–37)	**23%**	(10–37)	**22%**	(7–37)	**24%**	(10–38)	0.831
≤1,000	2,109	2,596	**77%**	(63–90)	**77%**	(63–90)	**78%**	(63–93)	**76%**	(62–90)	
**Previous TB**											
No	2,077	2,596	**80%**	(77–82)	**80%**	(77–82)	**11%**	(8–13)	**88%**	(87–90 )	**<0.001**
Yes	519	2,596	**20%**	(18–23)	**20%**	(18–23)	**89%**	(87–92)	**12%**	(10–13)	
**Regimen**											
d4T/AZT+3TC+NVP	2311	2,596	**89%**	(86–92)	**86%**	(86–92)	**22%**	(16–29)	**97%**	(95–99)	**<0.001**
d4T/AZT+3TC+EFV	244	2,596	**10%**	(8–12)	**8%**	(8–12)	**73%**	(66–80)	**2%**	(1–3)	
AZT/d4T+3TC+ABC	17	2,596	**1%**	(0–1)	**0%**	(0–1)	**4%**	(1–8)	**0%**	(0–1)	
Other	24	2,596	**1%**	(0–2)	**0%**	(0–2)	**0%**	(0–1)	**1%**	(0–2)	

Abbreviations: TB, tuberculosis; ART, antiretroviral therapy; IQR, inter-quartile range; CI, 95% confidence interval; WHO, World Health Organization; CTX, Co-trimoxazole; d4T, stavudine; AZT, zidovudine; 3TC, lamivudine; NVP, nevirapine; EFV, efavirenz; ABC, abacavir.

* Data in this column has been previously published [14].

** Compares characteristics of patients receiving (n = 271) and not receiving (n = 2,325) TB treatment at ART initiation.

Compared with patients not diagnosed as having prevalent TB at ART enrollment, patients with prevalent TB were more likely to be males (48% vs. 37%, p<0.001), have WHO stage III or IV disease (94% vs. 55%, p<0.001) (table 1), have weight <45 kg (34% vs. 16%, p<0.001), have a CD4 count <50/µL (26% vs. 15%, p = 0.001), have hemoglobin <8 g/dL (25% vs. 13%, p<0.001), and have been treated for TB prior to the current TB diagnosis (89% vs. 12%, p<0.001).

Efavirenz combined with lamivudine (3TC), and stavudine (d4T) or zidovudine (AZT), was prescribed to 73% of patients with active TB versus only 2% of patients assessed as TB-free (p<0.001) (table 1). In contrast, Nevirapine with 3TC, and d4T/AZT was prescribed to 97% of patients assessed as TB-free versus 22% of active TB patients at baseline (p<0.001).

**Figure 1:**
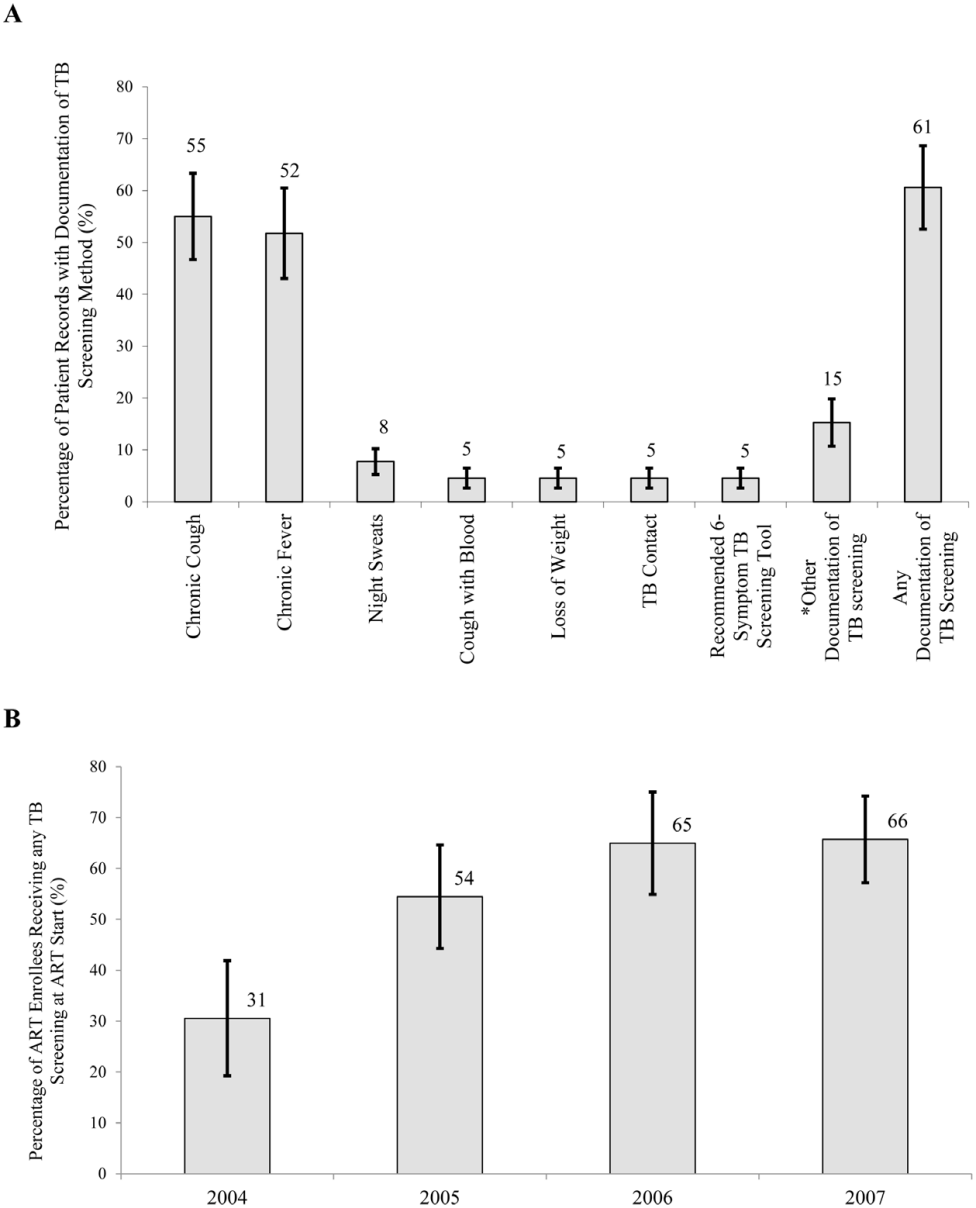
Compliance with TB Screening among ART Enrollees. **A** TB Screening among Patients at ART Enrollment in Mozambique during 2004–2007 (n = 2,596). **B** Improvement in Proportions of ART Enrollees Screened for TB in Mozambique during 2004–2007 (n = 2,596).

Among all ART enrollees, 61% (53–69%) had some documentation of TB screening in their medical records (figure 1A). The most common symptoms screened for were chronic cough (55%) and chronic fever (52%). Only 5% of ART enrollees during 2004–2007 were screened for all six recommended TB symptoms. Proportions of ART patients receiving some form of TB screening increased from 31% in 2004 to 66% in 2007 (p<0.001) (figure 1B).

**Figure 2:**
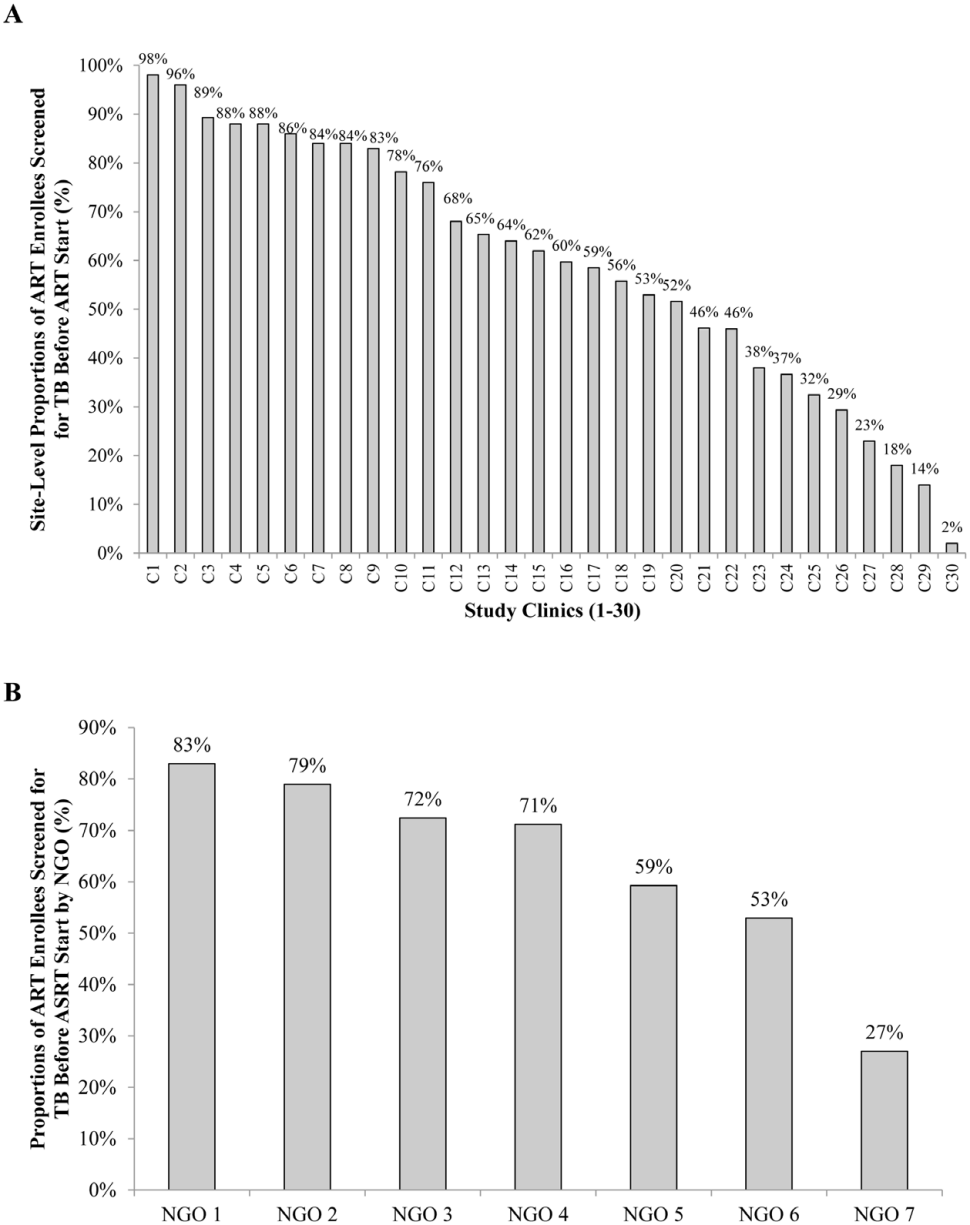
Compliance with TB Screening at ART Enrollment by Clinic and Supporting Non-Governmental Organization. **A** Compliance with TB Screening Guidelines According to ART Clinic (n = 2,596). **B** Compliance with TB Screening Guidelines According to Primary Non-Governmental Agency Supporting the ART Clinic (n = 2,596).

Proportions of ART enrollees with some documentation of TB screening in the records varied significantly by site, from 2% to 98% (p<0.001) (figure 2A). Larger sites [>1,000 patients (n = 12)] did not differ from smaller sites [<1,000 (n = 18)] in TB screening compliance (60% vs. 64%, p = 0.647) and rural sites (n = 9) did not differ from urban sites (n = 21) in TB screening compliance (67% vs. 58%, p = 0.188). However, all 30 clinics were supported by one of seven non-Governmental organizations (NGOs), and TB screening compliance was associated with the NGO providing support (figure 2B).

Only three patients not taking TB treatment at ART start [0.1% (95% CI, 0.0–0.2%)] were prescribed isoniazid preventive therapy (IPT).

**Figure 3:**
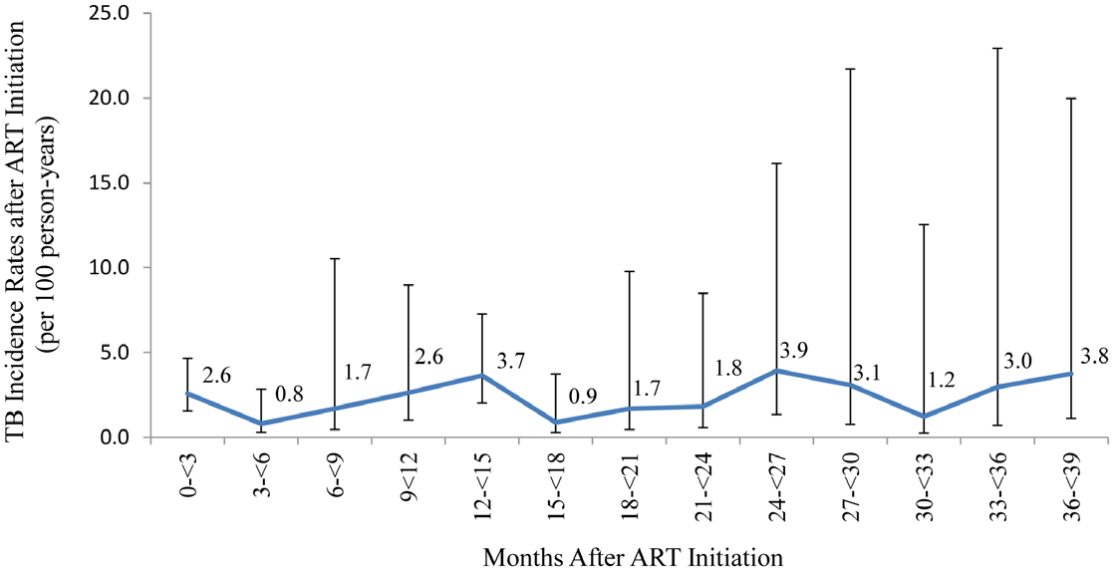
TB Incidence Rates during ART Follow-up among Patients Assessed as TB-free at ART Initiation (n = 2,325).

Among the 2,325 patients, who were considered TB-free at ART start by attending clinicians, 72 were diagnosed with pulmonary TB and 14 with extra-pulmonary TB during 3,438 person-years of ART follow-up, with an overall TB incidence rate of 2.32 cases per 100 person-years (95% CI, 1.80–3.05). TB incidence rates did not vary significantly across 3-monhtly ART follow-up intervals (figure 3).

**Figure 4:**
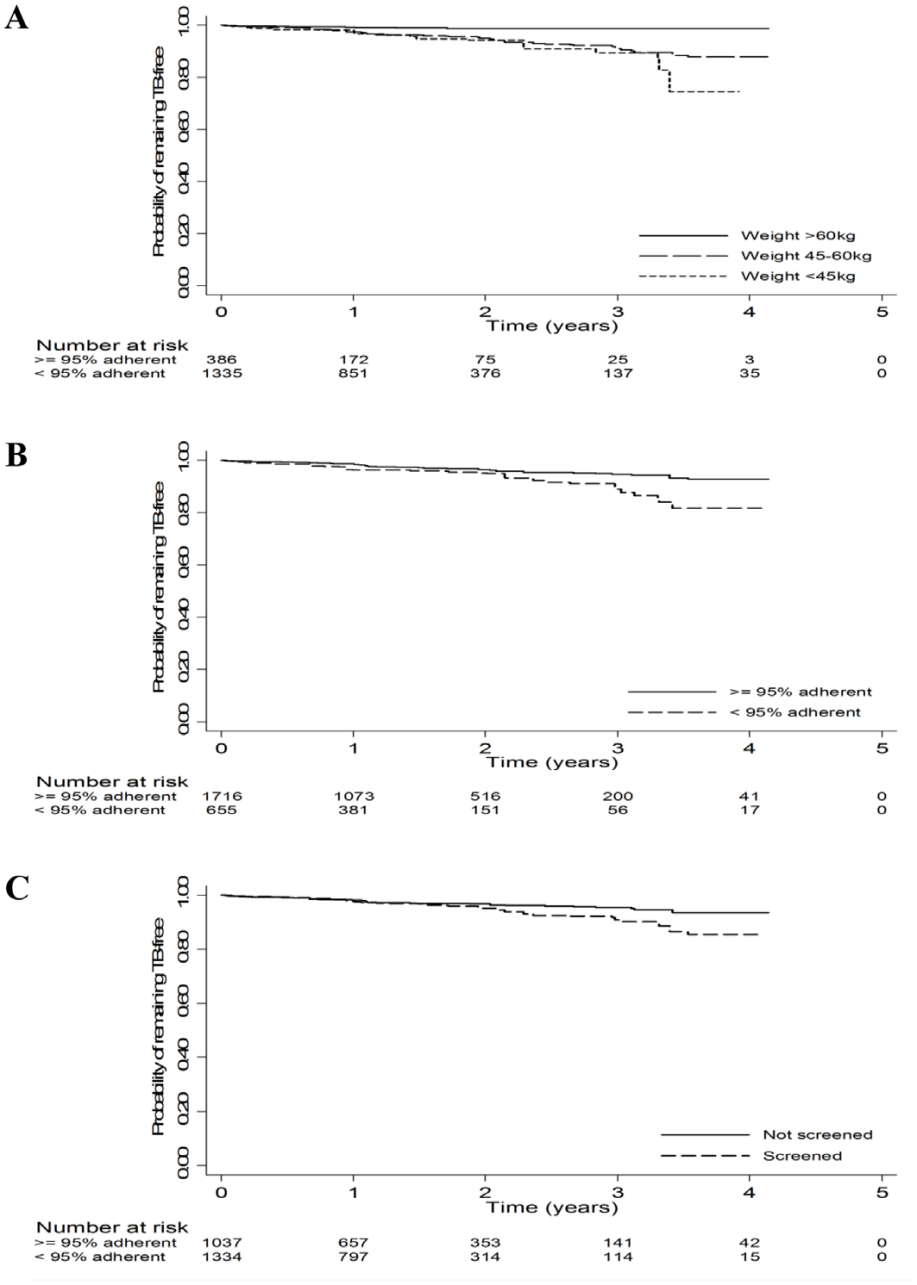
Kaplan Maier Analysis of TB-Free Survival. **A** Kaplan Meier Curve of TB Incidence Stratified by Baseline Weight. **B** Kaplan Meier Curve of TB Incidence Stratified by Level of ART Adherence. **C** Kaplan Meier Curve of TB Incidence Stratified by TB Screening at ART Start.

Baseline characteristics associated with increased risk of TB incidence included a 10-year age increase [adjusted hazards ratio (AHR) 1.40; 95% CI, 1.23–1.60] (table 2). Compared with employed patients, students had lower TB incidence (AHR 0.20; 95% CI, 0.07–0.55). Compared with patients having a weight >60kg at ART start, patients with a weight of 45–60 kg (AHR 2.52, 95% CI 0.91–7.01, p = 0.066) or <45 kg (AHR 3.50, 95% CI 0.84–14.54, p = 0.075) tended to have higher incidence of TB diagnosis (figure 4A). Similarly, compared with patients having a CD4>200/µL, patients with a CD4<50/µL tended to have higher TB incidence rates (AHR 1.79, 95% CI, 0.94–3.44, p = 0.074). Compared with patients assessed as having ART adherence >95%, patients with adherence <95% had higher TB incidence rates (AHR 2.06, 95% CI, 1.32–3.21) (figure 4B). Compared with patients for whom no documentation of TB screening at ART initiation was found, patients with documented TB screening at ART start had higher incidence of TB diagnosis during follow-up (AHR 1.60, 95% CI, 1.12–2.28) (figure 4C).

**Table 2:** Cox Proportional Hazards Analysis of Patient Characteristics Associated with TB Incidence during ART Follow-up.

		Original	Crude				Adjusted		
		No	Rate/100	HR	(95% CI)	p	AHR	(95% CI)	p
Age*		2,325	–	1.41	(1.25–1.58)	<0.001	1.40	(1.23–1.60)	<0.001
**Sex**									
Female		1,576	1.90	1.00			1.00		
Male		749	3.06	**1.59**	**(1.21–2.09)**	0.003	1.47	(0.95–2.27)	0.081
**Marital Status**									
Civil union|married		1,152	2.46	1.00			1.00		
Single|widowed		1,211	2.18	0.86	(0.60–1.22)	0.365	1.08	(0.71–1.63)	0.114
**Employment**									
Employed		992	2.80	1.00			1.00		
Student		107	0.36	**0.12**	**(0.05–0.30)**	**0.003**	**0.20**	**(0.07–0.55)**	**0.010**
Unemployed		1,169	2.04	0.76	(0.56–1.03)	0.072	0.90	(0.63–1.29)	0.536
**WHO Stage**									
Stage I/II		619	2.06	1.00			1.00		
Stage III		739	2.15	1.17	(0.64–2.12)	0.550	0.87	(0.41–1.85)	0.559
Stage IV		259	4.23	2.15	(0.88–5.20)	0.080	1.83	(0.55–6.05)	0.663
**Weight**									
>60 kg		470	1.17	1.00			1.00		
45–60 kg		1,224	2.63	2.35	(0.88–6.29)	0.073	2.52	(0.91–7.01)	0.066
<45 kg		367	3.64	3.16	(0.93–10.77)	0.061	3.50	(0.84–14.54)	0.075
**CD4 Count**									
≥200/µL		754	1.37	1.00			1.00		
50–<200/µL		1,144	2.60	1.68	(1.00–2.84)	0.052	1.45	(0.84–2.49)	0.165
<50/µL		356	3.55	**2.26**	**(1.21–4.23)**	**0.015**	1.79	(0.94–3.44)	0.074
**Hemoglobin**									
>8 g/dL		1,664	2.16	1.00			1.00		
<8 g/dL		235	3.60	1.79	(0.96–3.35)	0.065	1.40	(0.63–3.11)	0.365
**Co-trimoxazole**									
Prescribed CTX		821	2.55	1.00			1.00		
Not prescribed CTX		1775	2.21	0.82	(0.61–1.11)	0.180	0.98	(0.71–1.36)	0.919
**Adherence**									
≥95% adherent		1,263	1.92	1.00			1.00		
<95% adherent		597	3.54	**1.88**	**(1.29–2.73)**	**0.003**	**2.06**	**(1.32–3.21)**	**0.004**
**Site Size**									
>1,000		487	2.34	1.00			1.00		
≤1,000		2,109	2.24	0.93	(0.62–1.40)	0.700	0.75	(0.48–1.15)	0.170
**Previous TB**									
No		2,077	2.28	1.00			1.00		
Yes		519	2.59	1.15	(0.67–1.98)	0.591	0.89	(0.48–1.67)	0.708
**Screened at Baseline**									
Not screened		1,190	2.28	1.00			1.00		
Screened		1,406	2.59	**1.81**	**(1.23–2.67)**	**0.005**	**1.60**	**(1.12–2.28)**	**0.013**

Abbreviations: HR, hazards ratio; AHR, adjusted hazards ratio; TB, tuberculosis; CI, confidence interval; WHO, World Health Organization; CTX, Co-trimoxazole.

* Hazard ratio is reported for every 10 year increase in age at ART start.

## Discussion

This is the first nationally representative survey of TB screening, IPT uptake, and TB morbidity among enrollees in Mozambique’s national ART program and has several important findings. Firstly, the prevalence of TB at ART initiation and incidence of TB during ART follow-up were significantly higher than current estimates of TB prevalence and incidence in Mozambique’s general population [2]. Secondly, only 61% of ART enrollees had some documentation of TB screening, with TB screening compliance varying significantly by ART clinic and supporting NGO, suggesting that TB screening scale-up initiatives targeted at under-performing clinics and NGO partners, may be an efficient way to improve compliance with screening guidelines. Thirdly, IPT uptake was low with <1% of patients not taking TB treatment at ART initiation, receiving isoniazid. Fourthly, TB prevalence and incidence were associated with markers of advanced disease at ART initiation, suggesting that initiation of ART at earlier disease stages, could be an important TB prevention strategy [27]. Finally, patients with lower adherence to ART during the first 6 months of ART were more likely to develop incident TB during follow-up, suggesting that ART adherence interventions could help to reduce TB morbidity among ART patients.

### TB Burden

Our reported prevalence of active TB at ART start (11%) is 22 fold higher than that estimated for the general population (0.5%) [2]. The 11% prevalence is the same as that reported from Zambia [15], where national background TB incidence (566/100,000 in 2005 [2]), and median baseline CD4 count (143/µL) were similar. However, higher prevalence of TB among ART enrollees (21%) has been reported from South African settings where background TB incidence was higher (925/100,000 in 2005 [2]) and mean CD4 count at ART initiation lower (about 119/µL) [7]. Higher prevalence has also been reported among South African ART enrollees when TB culture was employed as a universal TB screening method; true TB prevalence rates of 15.5% [28], 17.3% [29], 25.7% [30], and 31.5% [31] have been reported in these settings. Our overall reported TB incidence rate of 2.32/100 person-years (95% CI, 1.80–3.05) is similar to that reported from routine program settings in Uganda (3.14/100 PY, 95% CI 2.82–3.49) [32], and Côte d’Ivoire (4.8/100 PY, 95% CI 2.5–8.3) [33], but slightly lower than that reported from South Africa (4.2/100 PY, 95% CI 3.8–4.5) [7]. Our reported TB incidence rate is about four-fold higher than that estimated for the general population in Mozambique in 2005 (554/100,000 population) [2].

Unlike other studies, TB incidence in our cohort did not decline with duration on ART [34]. Lack of decline in TB incidence rates during ART has been observed in ART cohorts where universal TB screening with TB culture prior to ART initiation has been implemented [31]. It is unlikely that intensive TB screening explains the lack of variation in TB incidence in our cohort, since only 5% of patients received the full six-symptom TB screen. One possible explanation is that undiagnosed symptomatic and sub-clinical TB present at ART initiation, which should have contributed to high early TB incidence rates during ART, went undiagnosed at the ART clinic, and may have contributed to high early rates of loss to follow-up (e.g. through hospitalization) or mortality [14].

### TB Screening and IPT Prescription

Formal TB screening criteria were not included in the national ART guideline document until 2007. In the same year, national efforts to increase TB screening compliance were implemented, especially by certain NGOs [13]. This probably explains the low prevalence of TB screening in 2004–2006, improvement in TB screening practices over time, and the association between TB screening and the NGO allocated to provide primary support for ART scale-up [13]. IPT for all HIV-infected patients in whom active TB has been excluded was only recommended by MOH in late 2006. This and the sub-optimal rates of TB screening at ART initiation likely explain the low rates of IPT prescription during 2004–2007. Another barrier to IPT scale-up may have been clinician concerns about isoniazid resistance. Even though available evidence suggests that the effect of IPT scale-up on isoniazid resistance is likely to be small [35], limited ability to definitively exclude active TB with available TB diagnostic tests (e.g. smear microscopy) and limited ability to monitor and ensure IPT adherence in a resource-constrained environment [36], may have hindered widespread IPT use [4]. Because both TB screening and IPT prescription for eligible patients at ART enrollment are important morbidity reduction and infection control interventions [37], uptake of these services should be monitored to assess continued improvement over time.

In our study, TB symptom screening at ART start was also associated with TB diagnosis and treatment during ART follow-up, with most differences in TB incidence noted after two years of follow-up (figure 4C). This could reflect the likelihood that clinicians who comply with TB screening guidelines at ART initiation will also screen for TB during long term ART follow-up [38], or it could reflect higher rates of LTFU or death among patients not screened for TB at ART start.

### Importance of Early ART

As in other studies, prevalence of TB at ART start was associated with markers of advanced disease (advanced WHO stage, previous TB, weight <45 kg, low CD4 count, and hemoglobin <8 g/dL) [7]. Similarly, markers of under-nutrition (weight<45 kg) and immune suppression (CD4<50/µL) tended to be associated with TB incidence during ART follow-up [5], [7], [32], [39]. Slower immune recovery during ART among older HIV-infected individuals [40], [41] may explain the higher rates of TB incidence in older ART patients. The association between TB and markers of advanced disease suggests that earlier treatment with ART, limiting time spent at low CD4 counts, could be an important TB prevention strategy for HIV-infected persons enrolled in care [27].

### Importance of ART Adherence

Poor adherence to antiretroviral pick-up appointments during months 0–6 of ART was associated with increased TB incidence. The poor adherence to pharmacy drug pick-up appointments could reflect higher risk for virological failure and slower immune recovery [17], [18], which has been associated with incident TB among ART cohorts in Europe and North America [38]. An alternative explanation for higher observed TB incidence among non-adherent patients, is that concurrent TB treatment caused poor adherence to antiretroviral drug pick-up appointments, however our own study showed no association between TB treatment at ART start and subsequent poor ART adherence, and other studies have reported no association between concurrent TB treatment and lower ART adherence [42]. As has been proposed by other authors, improving ART adherence could be an important intervention to reduce TB burden during ART [38].

### Limitations

This analysis has several limitations, including: (1) missing data on baseline covariates probably introduced non-differential measurement error, (2) we excluded patients with active TB at ART initiation from the time-to-event analysis; therefore, reported TB incidence rates may not be representative of these patients, (3) TB diagnostic results for some TB suspects identified at ART initiation may have returned after ART start, such that some reported incident TB may represent delayed diagnosis of prevalent TB at ART initiation, (4) follow-up time during later years of ART (e.g., years 2–4) may not be representative of patients enrolled during the latter parts of 2004–2007 because data were abstracted in November 2008, (5) because TB treatment clinics were usually run separately from ART clinics, some incident TB cases may not have been recorded in the ART medical records, and (6) similar to other studies [7], [32], [33] our study reports incidence of diagnosed and treated TB which may be an under-estimate of true TB incidence due to missed TB diagnoses (e.g. failure to identify TB suspects due to inconsistent TB screening during ART), or an over-estimate of true TB incidence due to incorrect initiation of TB treatment for some smear-negative TB suspects.

### Conclusions

The clinical burden of TB among ART enrollees was significant, increasing the importance of TB screening scale-up initiatives, IPT scale-up for adults screening negative, earlier ART to limit time spent at low CD4 counts, and adherence interventions for new ART enrollees.
